# To tolerate or to agree: A tutorial on tolerance intervals in method comparison studies with BivRegBLS R Package

**DOI:** 10.1002/sim.8709

**Published:** 2020-09-23

**Authors:** Bernard G. Francq, Marion Berger, Charles Boachie

**Affiliations:** ^1^ Technical R&D ‐ CMC Statistical Sciences GSK Rixensart Belgium; ^2^ Biostatistics and Programming, Non Clinical Biostatistics Sanofi Montpellier France; ^3^ Phastar Sheffield UK

**Keywords:** agreement, Bland‐Altman, BivRegBLS, coverage probabilities, method comparison studies, prediction interval, R package, tolerance interval

## Abstract

The well‐known agreement interval by Bland and Altman is extensively applied in method comparison studies. Two clinical measurement methods are considered interchangeable if their differences are not clinically significant. The agreement interval is commonly applied to assess the spread of the differences. However, this interval is approximate (too narrow) and several authors propose calculating a confidence interval around each bound. This article demonstrates that this approach is misleading, awkward, and confusing. On the other hand, tolerance intervals are exact and can include a confidence level if needed. Tolerance intervals are also easier to calculate and to interpret. Real data sets are used to illustrate the tolerance intervals with the R package BivRegBLS under normal or log‐normal assumptions. Furthermore, it is also explained how to assess the coverage probabilities of the tolerance intervals with simulations.

## 1 INTRODUCTION

In the 1980s, Bland and Altman started to publish papers dealing with the comparison of clinical measurement methods.^1^ Their paper published in *Lancet* in 1986 popularized their new concept of agreement intervals (AIs) (also known as limits of agreement).^2^ This paper is, 30 years later, the third most cited paper in statistics.^3^ Method comparison studies are conducted in many fields from engineering, cutting‐edge medicine, diagnostic tools, computing, robotic surgery, chemistry, but also in machine learning or artificial intelligence (with continuous outcome), and many other. Bland and Altman pinpointed through their papers the misuse of some erroneous statistical methods in this context of method comparison studies, that is, the Pearson correlation coefficient. They succeeded to convince researchers, clinicians, editors and journals to stop (or at least refrain from) the use of inappropriate statistics to assess the agreement of clinical measurement methods.^4^ The agreement interval then started to be widely applied, which launched a new research topic as many papers were published after that by many other authors including statisticians and clinicians (see the papers by John Ludbrook, surgeon, physiologist, and lastly biostatistician publishing papers related to method comparison studies).^5^, ^6^, ^7^, ^8^ The interval was, initially, an approximate solution to be used by medical researchers but, later, there was a need to improve it.^2^ Bland and Altman then proposed calculating a confidence interval around each bound of the approximate agreement interval. They proposed, first, an approximate solution which can be calculated quickly by hand.^2^ Later, they proposed a better solution using a more suitable standard deviation.^9^ An exact confidence interval around each bound of the approximate agreement interval has been recently proposed,^10^ or an approximate solution based on a resampling technique.^11^ This means that there is a need to improve the approximation of the agreement interval. However, while an exact interval can be directly calculated, this is not well known by practitioners, especially clinicians and researchers in medicine. This exact solution actually comes from the concept of *tolerance interval* (TI) in the statistical literature.

The tolerance intervals are not new, they were published in several papers in the 1940s, including by the famous statistician Wald,^12^, ^13^, ^14^ and papers from other authors over the following decades.^15^, ^16^, ^17^ A good overview and summary is given by Chew.^18^ These tolerance intervals are popular in industrial statistics.^19^ For instance, they can be calculated easily with the statistical software JMP (more commonly used in industry),^20^ SAS,^21^ or Minitab,^22^ while they can neither be calculated with the software MedCalc,^23^ nor NCSS that focuses on agreement intervals.^24^, ^25^ The reason that the tolerance intervals are not widely used in method comparison studies is to be questioned. In terms of terminology, tolerance means, in this context, that some difference between the methods is tolerated (the measurements are still comparable in practice). Furthermore, the tolerance interval is exact and therefore more appropriate than the agreement interval, as the next sections will show. Although many authors try to improve the approximation of the agreement interval or to calculate its confidence intervals,^11^, ^26^, ^27^, ^28^, ^29^ Ludbrook already pointed out the complexity of the agreement interval with its confidence intervals while the tolerance interval is the appropriate solution.^5^ Consequently, the tolerance interval should be recommended and promoted in this context of method comparison studies (also called bridging studies in pharmaceutical company). In more general viewpoint, tolerance intervals should be taught at school and part of the curriculum of statistical courses at universities as explained by Gitlow and Awad.^30^ Meeker et al^31^ also mention in their book that “Prediction intervals, in general, are no considered in most statistical texts except in the context of regression analysis.” This lack in the education of statisticians and engineers is probably the consequence of the nonuse of prediction and tolerance intervals in practice when they should be used.

The next section provides a nontechnical review and comparison of agreement and tolerance intervals. The new R package BivRegBLS^32^ will be used to illustrate the tolerance intervals and to compare erythrocyte measurements, aromatics concentration, or measures of fraction of unbound compounds. Full R code is given and it will be explained how to assess the coverage probabilities of the different statistical intervals by simulations.

## 2 TOLERANCE VS AGREEMENT INTERVALS

This section compares the agreement and tolerance intervals for unreplicated designs, where a given medical parameter (ie, blood samples, systolic or diastolic blood pressure, heart rate, oxygen saturation, etc) is measured for each patient (*n* patients) one time by both devices. The mathematical details for other designs can be found in the literature.^33^, ^34^, ^35^

Two clinical measurement methods are considered interchangeable if their (individual) differences are not clinically significant. The spread of the differences can be assessed by an interval within which 95% of the differences between the two clinical measurement methods are expected to lie. Note that this definition corresponds actually to a tolerance or prediction interval. The agreement interval by Bland and Altman was actually an attempt for a prediction interval, as explained in the next sections.

### 2.1 Agreement intervals

If $\bar{D}$ is the average of the differences and *S* the standard deviation of the differences, then the solution initially proposed by Bland and Altman is the 95% agreement interval (AI) where 95% of the differences are expected to lie:^9^

(1) $$\text{95\% AI:}\;\bar{D}\;\pm \;z_{0.975}S=\bar{D}\;\pm \;1.96S.$$

The quantity *z*_0.975_ refers to the 97.5*%* percentile of the standardized normal distribution and is equal to 1.96. This interval is approximate and is too narrow when the sample size is low as it does not take into account the sampling errors. Several authors propose to calculate a confidence interval around each bound of the AI.^9^, ^26^, ^27^, ^28^, ^29^ This approach aims to tackle the simplicity and the lack of accuracy of the agreement interval. The formula proposed by Bland and Altman is given as:

(2) $$\text{95\% CI for 95\% AI:}\;(\bar{D}\;\pm \;z_{0.975}S)\;\pm \;t_{0.975,n-1}S\sqrt{\frac{1}{n}+\frac{z_{0.975}^{2}}{2(n-1)}}.$$

The quantity *t*_0.975,*n* − 1_ refers to the 97.5% percentile of the Student's t‐distribution (with *n* − 1 degrees of freedom).


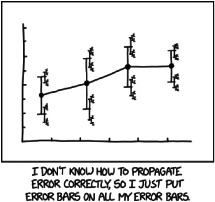
Errors Bars comic by Randall (2019)^36^

The agreement interval can then be replaced by an interval from the lower bound of the lower agreement limit to the upper bound of the upper limit. This interval can be interpreted as the worst case or worst scenario (the largest interval),^28^ even though there is no rationale to interpret it quantitatively. Calculating a confidence interval for each bound of the AI is even more complicated as it results in six values: the AI and a confidence interval on each of its bounds.

*Normal or t‐distribution?*

An analogy can be made between using the normal distribution or the Student's t‐distribution to calculate a confidence interval for a mean when the true variance is unknown. The latter results in an exact CI, while applying the normal distribution will result in an approximate CI that is also too narrow. Calculating a CI around each bound of the approximate CI for the mean would complicate the analysis, makes it awkward and confusing, and is useless as the exact CI with the t‐distribution is available. Just as the Student's t‐distribution is applied to calculate a CI for the mean, the TI should be applied to assess whether the differences between two clinical measurement methods are tolerable.

### 2.2 Prediction and tolerance interval

The prediction interval (PI) or equivalently beta‐expectation tolerance interval (βTI) provides an appropriate and exact solution that results in a single interval and it is therefore easier to calculate and to interpret. The 95% PI (or βTI) can be calculated as follows:^8^, ^34^

(3) $$\text{95\% TI:}\;\bar{D}\;\pm \;t_{0.975,n-1}S\sqrt{1+\frac{1}{n}}.$$

This interval is exact whatever the sample size and is not much more complicated to calculate than the AI (1) or (2).

The prediction interval here presented is equivalent to the beta‐expectation Tolerance interval (also called “tolerance interval type I,” see Table 1).^5^, ^18^, ^34^ Their definition and interpretation are, however, different. A prediction interval is an interval where a future measurement (eg, a future difference between two medical devices) is expected to lie with a given confidence level. A beta‐expectation tolerance interval is an interval where a given proportion of the population should lie, on average (eg, 95% of the differences). These two definitions lead to the same mathematical formula (3).

**TABLE 1:** Names and abbreviations of the agreement and tolerance intervals

Name	Abbreviation
Agreement interval	AI
Prediction interval	PI
Beta expectation tolerance interval	βTI or TI type I
Beta gamma content tolerance interval	βγTI, TI type II, or TI

### 2.3 Confidence in tolerance interval

The 95% beta‐expectation tolerance interval is one in which exactly 95% of the (individual) differences will lie, on average. This means that in practice it may be too narrow (in nearly 50% cases) or too large (in nearly 50% cases). It will contain exactly 95% of the differences on average when applied many times (infinite times). To improve this approach, a confidence level may be added to the tolerance interval. We can, for instance, calculate a 95% tolerance interval with an 80% confidence level. Such intervals contain at least 95% of the (individual) differences in 80% of cases. This confidence level provides an additional “guarantee” (a “margin of safety” or “assurance of safety”). These intervals are named “tolerance intervals of type II” or “beta‐gamma content tolerance intervals” (βγTI, see Table 1) and are given by the following formula:^34^

(4) $$\text{95\% TI with 80\% confidence:}\;\bar{D}\;\pm \;z_{0.975}S\sqrt{1+\frac{1}{n}}\sqrt{\frac{n-1}{\chi_{100\%-80\%,n-1}^{2}}}.$$

The quantity $\chi_{20\%,n-1}^{2}$ refers to the 20% percentile of the Chi square distribution (with *n* − 1 degrees of freedom). The normal distribution is related to the predictive level and the Chi square distribution is related to the confidence level. Note that the higher the confidence level, the larger the interval (the higher the assurance of safety).

This βγTI (or TI type II) cannot be obtained explicitly without the use of iterations. The formula presented in this article, (4), is an approximate formula derived from the first‐order Taylor approximation on the exact formula given by the following equations:^37^

(5) $$\bar{D}\;\pm \;kS,$$

where *k* is the solution of:

(6) $$\sqrt{\frac{2n}{\pi }}\int_{0}^{\infty }\mathrm{Pr}\left(\frac{\chi_{n-1}^{2}}{n-1}>\frac{\chi_{1,0.95,z^{2}}^{2}}{k^{2}}\right)e^{-\frac{1}{2}nz^{2}}dz=0.8,$$

where $\chi_{1,0.95,z^{2}}^{2}$ is the 95% percentile of the Chi square distribution with one degrees of freedom and a noncentrality parameter equal to *z*^2^. Comparing (4) with (3), one can notice that the Student distribution is replaced by a normal distribution and the standard deviation by the upper bound of its confidence interval.^37^ This leads to an explicit formula easy to calculate with excellent predictive and confidence levels as shown in the next section, with similar results in practice.^10^, ^16^, ^31^, ^34^

#### 2.3.1 Prediction, tolerance, and quantiles? One or two‐sided?

The statistical intervals presented in this article are two‐sided. The formulas for confidence and prediction intervals are usually identical for one‐ or two‐sided versions except that the “−” or “+” sign has to be chosen accordingly for lower or upper one‐sided version and the risk associated with the confidence level has to be divided by 2 for the two‐sided version (left and right tails). The mathematical formulae for the one‐sided or the two‐sided βγTI are, however, not the same. The exact one‐sided TI requires a noncentral t distribution (not shown in this article).

A one‐sided beta‐gamma content tolerance interval is, actually, equivalent to calculating a confidence interval for a given quantile, that is, the upper one‐sided 80% CI for the quantile 0.95 is identical to the upper one‐sided 95% TI (type II) with 80% confidence level.^31^ On the other hand, the two‐sided βγTI are not equivalent to calculating confidence intervals on two quantiles (ie, 80% CIs on quantiles 0.025 and 0.975).^37^ If one calculates the lower and the upper one‐sided βγTI, both at 97.5% with 80% confidence levels, then the interval obtained from these two bounds would be an approximate two‐sided 95% TI with 80% confidence levels.^31^ The exact two‐sided TI (6) or its direct approximation (4) are better.

Note that the distinction between enumerative and analytic studies was introduced by Deming,^38^ and further discussed by Meeker et al.^31^ The comparison of two manufacturing processes, the comparison of drugs, or the comparison of machines are examples given where we want to draw inferences or make predictions about the future (which is the goal of the prediction or tolerance interval type II). These example are common in engineering or medical fields.^31^ In our context, clinicians have to make decisions about the performance of the new analytical method to be used in the future based on data obtained in the past.

#### 2.3.2 Bayesian statistics and resampling methods

Under the Bayesian framework, prediction intervals can be directly calculated from the posterior distribution, while tolerance intervals (type II) can be obtained after some extracalculation (an iterative search). Bayesian tolerance intervals in the univariate normal distribution are discussed by Krishnamoorthy and Mathew^37^ ^(section 11.2)^, under the noninformative prior and under the conjugate prior. This Bayesian methodology can be extended and applied to method comparison studies as the differences between two analytical methods provide a univariate distribution, assumed to be normal. Prediction intervals can also be obtained by bootstrap techniques. The data are resampled, the mean and the standard deviations are calculated from the pseudo‐sample, then the pivotal root (derived from (3)) is calculated at each bootstrap simulation. The quantile of the t‐distribution in (3) can, then, be replaced by the quantiles from this bootstrap pivotal root distribution to obtain a bootstrap‐t prediction interval. The coverage probabilities of this resampling method are slightly lower than the nominal level for small sample sizes.

### 2.4 Coverage probabilities of the agreement and tolerance intervals

**FIGURE 1:**
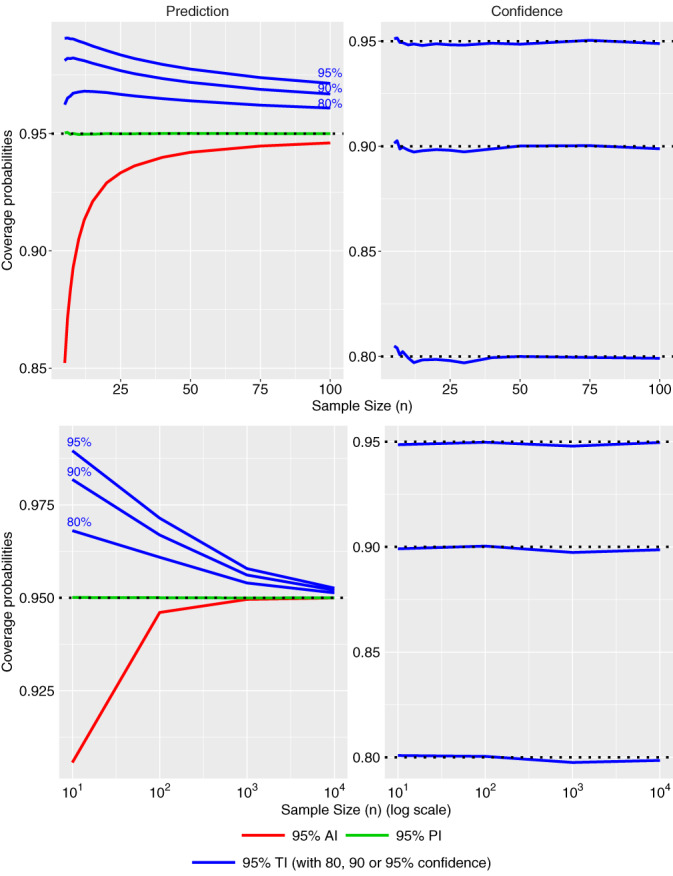
Predictive levels (left) of the 95% agreement interval (AI), the 95% tolerance interval (TI) type I and the 95% TI type II with 80%, 90%, or 95% confidence levels, and the corresponding confidence levels of the TI type II (right), according to the sample size (n) from 5 to 100 (top), and from 10^1^ to 10^4^ (bottom) [Colour figure can be viewed at wileyonlinelibrary.com]

In order to compare the effective prediction (and confidence) levels of the agreement and tolerance intervals, 10^6^ samples were simulated for different sample sizes (number of patients) from *n* = 5 to *n* = 100 (and from *n* = 10^1^ to *n* = 10^4^) with an unreplicated design and under equivalence (one measure per patient and per measurement method; the measurements are equal before adding a random Gaussian measurement error to both measurements). The standard deviation of the measurement errors are set to 1 for both devices (X and Y), the prediction level to 95% and the confidence levels for the βγ TI (TI type II) are set to 80%, 90%, and 95%. For each simulated set of data, the mean and the standard deviation of the differences are estimated, and the different intervals are calculated. The effective prediction levels are then calculated based on a normal distribution with mean 0 and standard deviation equal $\sqrt{1+1}=1.414$. After the 10^6^ simulations, the average of the effective prediction levels is calculated for each interval and displayed in Figure 1 (left). The confidence levels of the βγTIs are also assessed and displayed in Figure 1 (right). R code is given in the Appendix.

Figure 1 (left) illustrates the effective prediction levels of the 95% AI, 95% βTI, and 95% βγTI with 80%, 90%, or 95% confidence level, according to the sample size (number of patients). The effective prediction level of the AI is always lower than the nominal level and it collapses for small sample sizes. The effective prediction level of βTI is exactly equal to the nominal level whatever the sample size (the values are given in the Appendix). The effective predictive level of βγTI is higher than the nominal level as the “assurance of safety” enlarges the interval. Figure 1 (right) illustrates that the effective confidence levels of the βγTIs are equal to the targeted confidence level, whatever the sample size. Figure 1 (bottom) shows the convergence of the different intervals from small to big sample size. The AI converges to the PI (βTI) for, approximately, *n* = 10^3^, while the βγTI converges to the PI, approximately, for *n* = 10^4^.

## 3 APPLICATIONS USING BIVREGBLS R PACKAGE

The tolerance intervals can be calculated with the free statistical software R (downloadable from the website of R‐CRAN).^39^ Tolerance^40^ is a very good R package to calculate tolerance intervals under a wide variety of distributions or models. BlandAltmanLeh^41^ is an R package to calculate the agreement intervals, also blandr^42^ to calculate their confidence intervals but this is not recommended as explained through this article.

The R package BivRegBLS^32^ is specifically designed to calculate tolerance intervals in method comparison studies and to estimate errors‐in‐variables regressions with many different statistical intervals. An updated version has been recently published on CRAN.^43^ Additional details and R command lines are given in the next sections to calculate tolerance intervals in an (M,D) plot.

### 3.1 MD.horiz.lines and MD.plot R functions

The function MD.horiz.lines can be used to calculate the tolerance intervals in method comparison studies in an (M,D) plot (arithmetic means on *x*‐axis and difference on *y*‐axis). The function has five arguments:


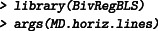



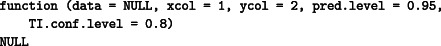


First, the data set to be used. Second (third), the column name with the x (y) measures (the numeric position of the column can also be used). Fourth, the prediction level to be used for the tolerance intervals (default value is 0.95). Fifth, the confidence level to be used for the βγ content tolerance interval. The results can be visualized in an (M,D) plot by using the function MD.plot where additional arguments (eg, axis limits, axis titles, main title) can be used as an ellipsis is implemented:


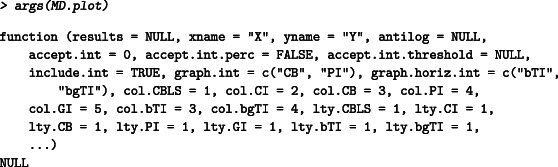


The *results* must be from the CBLS function (not shown in this article) or the MD.horiz.lines, *xname* and *yname* can be used as a character string to name, respectively, the X and Y devices.

The *antilog* argument can be used to back‐transform the results if a log scale is used (see next section). The acceptance interval can be defined using the argument *accept.int*: a numeric vector (length equal to 1 or 2) for the value of Δ to visually check that |Y−X|<Δ. Two values of Δ can be used in case the acceptance interval changes for low or high values by using the argument *accept.int.threshold* (see example in next section). The acceptance interval can be used in percentage by setting the argument *accept.int.perc* as TRUE.

By default, the two tolerance intervals are displayed on the (M,D) plot, but the user can specify which one to plot with the argument *graph.horiz.int* (“bTI” for the beta‐expectation TI and/or “bgTI” for the beta‐gamma content TI). The line type and color of the tolerance intervals can be changed using the arguments *col.bTI, col.bgTI, lty.bTI, lty.bgTI*.

### 3.2 Differences vs means—Absolute differences

A data set kindly provided by Sanofi is used to illustrate and compare the different intervals. Hematology measurements are performed on 20 dogs with two devices: A120 and TH1. The erythrocyte measurements (in $10^{6}/\mu$L) are given as follows:


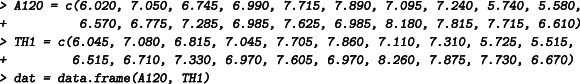


The average of the differences, *D* = A120 − TH1, can then be calculated as $\bar{D}=-0.012$, and the standard deviation as *S* = 0.047. The 95% AI can then be calculated as follows:

$$\bar{D}\;\pm \;1.96S=-0.012\;\pm \;1.96\cdot 0.047=[-0.103,0.080].$$

The AI should be interpreted with caution, as it is approximate and too narrow. One could say that less of 95% of the differences lie approximately between −0.103 ($10^{6}/\mu$L) and 0.080 ($10^{6}/\mu$L). By using formula (2), one could calculate the 95% CIs around each bound as:

$$(\bar{D}\;\pm \;z_{0.975}S)\;\pm \;t_{0.975,19}S\sqrt{\frac{1}{n}+\frac{z_{0.975}^{2}}{2(n-1)}}=\{-0.103[-0.141,-0.065];0.080[0.042,0.117]\}.$$

The interval in the worst case is therefore [−0.141, 0.117] ($10^{6}/\mu$L). This interval cannot be interpreted correctly. The 95% PI or 95% βTI can be calculated as follows:

$$\bar{D}\;\pm \;t_{95\%,19}S\sqrt{1+1/n}=-0.012\;\pm \;2.093\cdot 0.047\sqrt{1+1/20}=[-0.112,0.088].$$

This interval can be directly interpreted as: a future difference between the two devices (A120 and TH1) is expected to lie between −0.112 and 0.088 (with 95% confidence) (prediction interval interpretation), or exactly 95% of the future differences will lie between −0.112 ($10^{6}/\mu$L) and 0.088 ($10^{6}/\mu$L), on average (βTI interpretation).

If needed, a confidence level can be added to the beta‐expectation tolerance interval by calculating the βγ content TI. For instance, the 95% TI with 80% confidence level is given by:

$$\bar{D}\;\pm \;1.96S\sqrt{1+1/n}\sqrt{\frac{n-1}{\chi_{20\%,19}^{2}}}=-0.012\;\pm \;1.96\cdot 0.047\sqrt{1+1/20}\sqrt{\frac{19}{13.716}}=[-0.122,0.098].$$

The interpretation is: at least 95% of the future differences will lie between −0.122 ($10^{6}/\mu$L) and 0.098 ($10^{6}/\mu$L) in 80% of cases (and in 20% of the cases, less than 95% of the future differences will lie in this interval).

Note that a βγTI (type II) with 95% predictive level and 95% confidence level may provide a too large interval in practice but the confidence level has to be determined a priori according to the study objectives. The main advantage of the βγTI over the βTI is that it controls the risk of falling outside the interval. We are confident in what we get (we “control”), which is not the case for βTI. Note that a 95% βTI is similar (but not exactly equal) to a 95% βγTI with 50% confidence level.^34^

**FIGURE 2:**
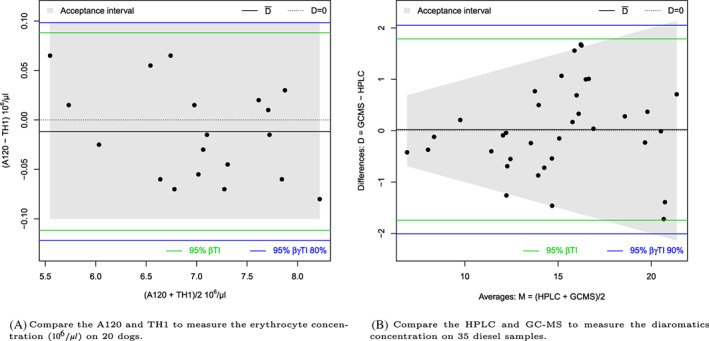
Mean‐difference (Bland‐Altman) plot with tolerance intervals [Colour figure can be viewed at wileyonlinelibrary.com]

The tolerance intervals are illustrated in Figure 2A where the mean‐difference (Bland‐Altman) plot is produced (the differences are plotted on the *y*‐axis against the averages on the *x*‐axis). We can notice that the βγTI is larger than the βTI as a confidence level is added (the safety margin). If we assume that differences up to 0.1 ($10^{6}/\mu$L) are not clinically important, it means that we accept or tolerate differences up to 0.1 ($10^{6}/\mu$L). The acceptance interval is then defined as [− 0.1,0.1] ($10^{6}/\mu$L). In this case, whatever the chosen interval, there is insufficient evidence to conclude equivalence (both TIs are larger). One can conclude that the A120 and TH1 are not “equivalent” and therefore not interchangeable (when measuring the erythrocyte concentration).

The function MD.horiz.lines can be used to calculate the tolerance intervals as follows:


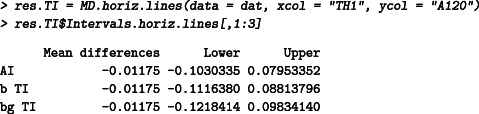


The results can be visualized in an (M,D) plot, see Figure 2A by using the function MD.plot as follows:


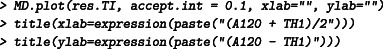


Note that the normality is assumed to calculate the statistical intervals in this paper. The *P*‐value of the Shapiro‐Wilk test for normality on the differences A120 − TH1 is 0.2.

### 3.3 Differences vs means—Relative acceptance interval

In Ferrer et al,^44^ the monoaromatics, diaromatics, and triaromatics concentrations of 35 diesel samples are measured by HPLC and GC‐MS (the results are presented in weight percentage). The full data set is included in BivRegBLS.

#### 3.3.1 Univariate tolerance intervals

For illustration, 95% βTI and 95% βγTI with 90% confidence are calculated on the diaromatics concentrations, and an acceptance interval of 10% is used. The higher the measure, the higher the acceptance interval. For example, 1 unit difference is tolerated for average measures of 10, and 2 units of differences is tolerated for average measures of 20. The results can be visualized in Figure 2B. A future difference between the HPLC and GC‐MS is expected to lie between −1.74 and 1.79 (with 95% confidence), or equivalently 95% of the differences are expected to lie between these two values (on average). In 90% of cases, at least 95% of the differences will lie between −2.01 and 2.05. The *P*‐value of the Shapiro test is .79.


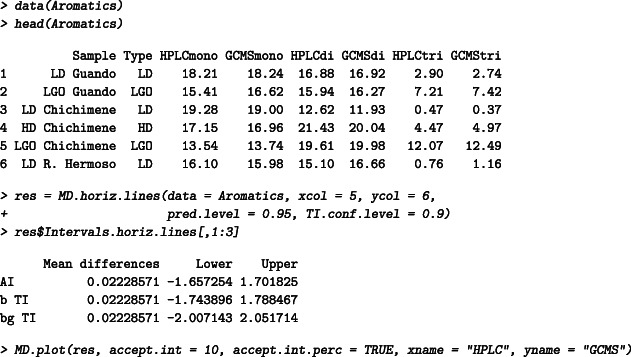


#### 3.3.2 Multivariate tolerance intervals

If a 95% βγTI with 90% confidence level is calculated for each of the three concentration parameters (monoaromatics, diaromatics, and triaromatics), then the confidence level is to be interpreted for each interval separately. If needed, the confidence level can be adjusted (like in an ANOVA) so that, the first TI (for monoaromatics concentrations) and the second TI (for diaromatics concentrations) and the third TI (for triaromatics concentrations) will contain, each, at least 95% of their respective differences between HPLC and GC‐MS in 90% of cases. Šidák adjustment is a straightforward approach. In this case, each TI will be calculated with 0.9^1/3^ = 96.55*%* confidence level, such that, overall, the confidence level will be 90%. A better approach is, maybe, the use of multivariate tolerance intervals,^37^ but the main disadvantage is the difficulty to communicate the results to nonstatisticians as multivariate tolerance regions are, usually, given by an ellipse, or an ellipsoid in this example. See also the paper by Fuchs and Kenett^17^ for multivariate tolerance regions and its connection to the F‐test.

### 3.4 Ratios vs geometric means—Log normal

**FIGURE 3:**
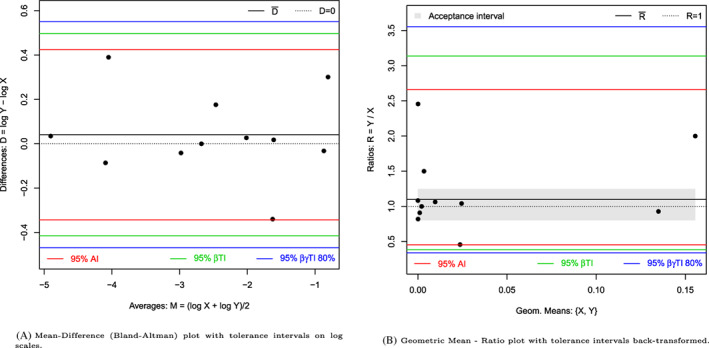
Comparison between the flux dialysis and reported values on fraction unbound of 11 compounds [Colour figure can be viewed at wileyonlinelibrary.com]

The data published by Kalvass et al^45^ is used to illustrate the tolerance intervals under log normal distributed data. The fraction unbound of highly bound compounds is measured on 11 compounds with a flux dialysis technique and then compared with the values given in the literature for each of these compounds. A logarithmic transformation is applied and the tolerance intervals are calculated, see Figure 3A. The acceptance interval cannot be properly defined on log scales. The results are then back‐transformed using the function antilog.pred with a base 10. To plot the back‐transformed statistical intervals, the user can use directly the function MD.plot with the argument antilog. With the back‐transformation, the plot is automatically created with geometric means on the *x*‐axis and ratios on the *y*‐axis, see Figure 3B. It is here assumed that differences of 20% are not meaningful. The acceptance interval starts then at (1 − 0.2 = 0.8) and ends at 1/(1 − 0.2) = 1.25.


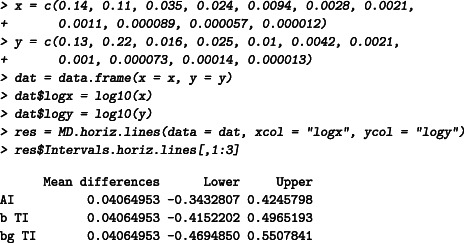



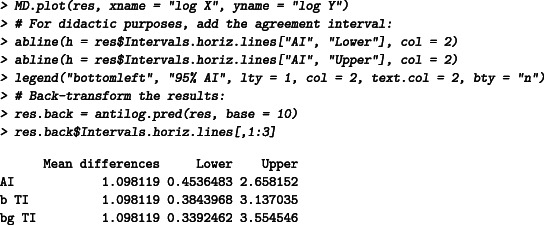



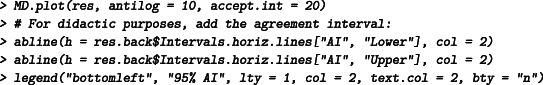


The flux dialysis is expected to differ from 0.38‐ to 3.1‐fold from the values reported by the literature (with 95% confidence) (prediction interval interpretation), or exactly 95% of the future measurements will differ from 0.38‐ to 3.1‐fold, on average (βTI interpretation). If needed, a confidence level can be added by calculating the βγTI (type II). The 95% TI with 80% confidence level is given by [0.34,3.55]: at least 95% of the future flux dialysis measures will differ from 0.34‐ to 3.55‐fold from the reported values in 80% of cases. Note that the agreement interval is given in the output of the function MD.horiz.lines for “didactic” reasons but cannot be plotted automatically by the MD.plot function. In Figure 3A,B, we have added the agreement interval to illustrate that the narrowness of the agreement interval is highlighted when the interval is exponentiated to back‐transform the results from a log scales to the original scales. The *P*‐value of the Shapiro test, on log scale, is .43.

### 3.5 Tolerance intervals with repeated measures

When repeated measures are available, the function MD.horiz.lines can still be used and the results plotted with the function MD.plot. The repeated measures should be in an unstacked format (different columns) and the argument xcol and ycol should define these columns (eg, xcol = 1:3, or xcol = c(“R1,” “R2,” “R3”)).

### 3.6 Estimate a regression line

The tolerance intervals presented in this article are bounded by two straight (horizontal) lines in the mean‐difference (Bland‐Altman) plot and are therefore valid if no pattern is observed (or if at least, the differences can still be assumed to be Gaussian). Otherwise, a regression line should be estimated by the correlated bivariate least square (CBLS) regression or equivalently by the bivariate least square (BLS) regression in the (X,Y) plot.^34^, ^46^ The functions BLS and CBLS are included in BivRegBLS.

### 3.7 Validation of BivRegBLS

The first release of BivRegBLS on CRAN (version 1.0.0) contains 18 functions including five graphical functions, with more than 160 arguments in total. More than 200 warning and error messages are implemented to help the user. The full package has been validated with more than 550 user acceptance tests (UAT) and more than 160 user technical tests (UTT).

## 4 RECOMMENDATIONS AND CONCLUSION

### 4.1 Are the tolerance intervals more complicated to calculate than the agreement intervals?

The tolerance intervals can be calculated with a single formula and they are not much more complicated than the agreement interval. Moreover, trying to improve the approximation of the agreement interval leads to more complicated formulas than directly calculating the exact beta‐expectation tolerance interval.

### 4.2 Are the tolerance intervals more complicated to interpret than the agreement intervals?

The beta‐expectation tolerance interval is easier to interpret as it contains exactly the desired predictive level (on average). The beta‐gamma content tolerance interval is also easy to interpret as it encompasses a given proportion of the data with a given confidence level. The agreement interval is approximate and therefore, it should be interpreted with caution (especially for small sample sizes). Calculating a confidence interval for each bound of the agreement interval is awkward and brings even more confusion to its interpretation. While Hamilton and Stamey recommend calculating the AIs with its confidence intervals in their paper entitled “Using Bland‐Altman to assess agreement between two medical devices‐don't forget the confidence intervals!,”^26^ we have shown in this article that tolerance intervals are better and should be encouraged.

### 4.3 Tolerance intervals with BivRegBLS?

The functions MD.horiz.lines is straightforward to calculate the tolerance intervals with only three main arguments: the data set, the *X* column, and the *Y* column, while additional arguments can be used to change the predictive or confidence levels of the tolerance intervals. The function MD.plot allows to quickly visualize the results and the tolerance intervals. A constant or relative acceptance interval can be added to the plot. Finally, the results can easily be back‐transformed when a logarithmic transformation is applied. The plot is then converted accordingly with geometric means and ratios instead of the traditional mean‐difference (Bland‐Altman) plot.

## CONFLICT OF INTERESTS

B.G.F. and C.B. were employees of the University of Glasgow when writing the first draft of this manuscript. B.G.F. is an employee of the GSK group of companies, M.B. is an employee of Sanofi, and C.B. is an employee of Phastar; there is no business relationship between these companies.

## AUTHOR CONTRIBUTIONS

The literature search was performed by B.G.F., the R code and plots by B.G.F., the applications were chosen by B.G.F. and M.B. First draft of the manuscript by B.GF., then with C.B. All authors were involved in drafting the final version of the manuscript and all authors critically revised it for important intellectual content. All authors approved the manuscript before it was submitted by the corresponding author.

5

## Data Availability

n/a

## APPENDIX 1

### 1.1

The full R code to assess the coverage probabilities of the tolerance intervals (Figure 1, top) is given as follows:

## References

[1] Altman DG , Bland JM . Measurement in medicine: the analysis of method comparison studies. Statistician. 1983;32:307‐317.

[2] Bland JM , Altman DG . Statistical methods for assessing agreement between two methods of clinical measurement. Lancet. 1986;327(i):307‐310. 2868172

[3] Van Noorden R , Maher B , Nuzzo R . The top 100 papers. Nature. 2014;514:550‐553. 25355343 10.1038/514550a

[4] Elsevier. https://www.elsevier.com/journals/physiotherapy/0031‐9406/guide‐for‐authors. Accessed July 03, 2016.

[5] Ludbrook J . Confidence in Altman‐Bland plots: a critical review of the method of differences. Clin Exp Pharmacol Physiol. 2010;37:143‐149. 19719745 10.1111/j.1440-1681.2009.05288.x

[6] Ludbrook J . Linear regression analysis for comparing two measurers or methods of measurement: But which regression? Clin Exp Pharmacol Physiol. 2010;37:692‐699. 20337658 10.1111/j.1440-1681.2010.05376.x

[7] Ludbrook J . Comparing methods of measurement. Clin Exp Pharmacol Physiol. 1997;24:193‐203. 9075596 10.1111/j.1440-1681.1997.tb01807.x

[8] Ludbrook J . Statistical techniques for comparing measurers and methods of measurement: a critical review. Clin Exp Pharmacol Physiol. 2002;29:527‐536. 12060093 10.1046/j.1440-1681.2002.03686.x

[9] Bland JM , Altman DG . Measuring agreement in method comparison studies. Stat Methods Med Res. 1999;8:135‐160. 10501650 10.1177/096228029900800204

[10] Carkeet A . Exact parametric confidence intervals for Bland‐Altman limits of agreement. Optom Vis Sci. 2015;92(3):135‐160. 25650900 10.1097/OPX.0000000000000513

[11] Parker RA , Weir CJ , Rubio N , et al. Application of mixed effects limits of agreement in the presence of multiple sources of variability: exemplar from the comparison of several devices to measure respiratory rate in COPD patients. PLoS One. 2016;11(12):1‐15. 10.1371/journal.pone.0168321. PMC5156413 27973556

[12] Wald A . Setting of tolerance limits when the sample is large. Ann Math Stat. 1942;13:389‐399.

[13] Wald A . An extension of Wilk's method for setting tolerance limits. Ann Math Stat. 1943;14:45‐55.

[14] Wald A , Wolfowitz J . Tolerance limits for a normal distribution. Ann Math Stat. 1946;17(2):208‐215.

[15] Wilks SS . Statistical prediction with special reference to the problem of tolerance limits. Ann Math Stat. 1942;13:400‐409.

[16] Howe WG . Two‐sided tolerance limits for normal populations, some improvements. J Am Stat Assoc. 1969;64(326):610‐620.

[17] Fuchs C , Kenett RS . Multivariate tolerance regions and F‐tests. J Qual Technol. 1987;19(3):122‐131.

[18] Chew V . Confidence, prediction, and tolerance regions for the multivariate normal distribution. J Am Stat Assoc. 1966;61(315):605‐617.

[19] Kenett RS , Zacks S . Modern Industrial Statistics with Applications in R, MINITAB and JMP. 2nd ed. New York, NY: Wiley; 2014.

[20] JMP . JMP®, Version 14 1989‐2007. Cary, NC: SAS Institute Inc; 2019.

[21] SAS . Base SAS® 9.4 Procedures Guide. Cary, NC: SAS Institute Inc; 2019.

[22] Minitab . Minitab® v. 17. State College, PA: Minitab, Inc; 2019.

[23] MedCalc . MedCalc Statistical Software version 16.4.3. Ostend, Belgium: MedCalc Software bvba; 2016.

[24] NCSS . NCSS, Statistical Software. Kaysville, Utah: NCSS, LLC; 2019 ncss.com/software/ncss.

[25] NCSS https://www.ncss.com/software/ncss/method‐comparison‐in‐ncss/. Accessed November 01, 2019.

[26] Hamilton C , Stamey J . Using Bland‐Altman to assess agreement between two medical devices–don't forget the confidence intervals! J Clin Monit Comput. 2007;21(6):331‐333. 17909978 10.1007/s10877-007-9092-x

[27] Olofsen E , Dahan A , Borsboom G , Drummond G . Improvements in the application and reporting of advanced Bland‐Altman methods of comparison. J Clin Monit Comput. 2015;29(1):127‐139. 24806333 10.1007/s10877-014-9577-3

[28] Bland JM 2001 https://www‐users.york.ac.uk/∼mb55/talks/rcrtalk.htm. Accessed September 03, 2016.

[29] Giavarina D . Understanding Bland Altman analysis. Biochem Med. 2015;25(2):141‐151. 10.11613/BM.2015.015 PMC4470095 26110027

[30] Gitlow H , Awad H . Intro stats students need both confidence and tolerance (Intervals). Am Stat. 2013;67(4):229‐234. 10.1080/00031305.2013.839482.

[31] Meeker WQ , Hahn GJ , Escobar LA . Statistical Intervals: A Guide for Practitioners and Researchers. 2nd ed. New York, NY: Wiley; 2017.

[32] Francq Bernard G , Berger Marion . BivRegBLS: Tolerance Intervals and Errors‐in‐Variables Regressions in Method Comparison Studies R package version 1.0.0 2017.

[33] Choudhary PK . A tolerance interval approach for assessment of agreement in method comparison studies with repeated measurements. J Stat Plann Inference. 2008;138:1102‐1115.

[34] Francq BG , Govaerts B . How to regress and predict in a Bland‐Altman plot? review and contribution based on tolerance intervals and correlated‐errors‐in‐variables models. Stat Med. 2016;35:2328‐2358. 26822948 10.1002/sim.6872

[35] Vidmar G , Burger H , Erjavec T . Options for comparing measurement agreement between groups: exercise testing as screening for ability to walk after transfemoral amputation. Informatica Medica Slovenica. 2010;15(2):10‐20.

[36] Randall Munroe . https://www.explainxkcd.com/wiki/index.php/2110:_Error_Bars. Accessed May 10, 2019.

[37] Krishnamoorthy K , Mathew T . Statistical Tolerance Regions: Theory, Applications, and Computation. New York, NY: Wiley; 2009.

[38] Deming WE . On the distinction between enumerative and analytic surveys. J Am Stat Assoc. 1953;48:244‐255.

[39] R Core Team . R: A Language and Environment for Statistical Computing. Vienna, Austria: R Foundation for Statistical Computing; 2019.

[40] Young DS . tolerance: an R package for estimating tolerance intervals. J Stat Softw. 2010;36(5):1‐39.

[41] Lehnert Bernhard . BlandAltmanLeh: Plots (Slightly Extended) Bland‐Altman Plots. R Package Version 0.3.1; 2015.

[42] Datta Deepankar . blandr: a Bland‐Altman Method Comparison Package for R; 2017.

[43] Francq Bernard G , Berger Marion . BivRegBLS: Tolerance Interval and EIV Regression ‐ Method Comparison Studies, R Package Version 1.1.1 2019.

[44] Baldrich C , Murcia B , Bueno A . Development of a methodology to determine the aromatic structural distribution in light and medium petroleum fractions by HPLC. CT&F Ciencia, Tecnologïa y Futuro. 2006;3(12):149‐162.

[45] Kalvass J , Cory PC , Jenkins Gary J , et al. Mathematical and experimental validation of flux dialysis method: an improved approach to measure unbound fraction for compounds with high protein binding and other challenging properties. Drug Metab Dispos. 2018;46(4):458‐469. 10.1124/dmd.117.078915. 29437872

[46] Francq BG , Govaerts BB . Measurement methods comparison with errors‐in‐variables regressions. From horizontal to vertical OLS regression, review and new perspectives. Chemom Intell Lab Syst. 2014;134:123‐139.

